# Safety and Efficacy of Programmed Cell Death 1 and Programmed Death Ligand-1 Inhibitors in the Treatment of Cancer: An Overview of Systematic Reviews

**DOI:** 10.3389/fimmu.2022.953761

**Published:** 2022-07-13

**Authors:** Shun-Long Ou, Jing Luo, Hua Wei, Xiao-Li Qin, Su-Ya Du, Song Wang, Qian Jiang

**Affiliations:** ^1^ Department of Pharmacy, Sichuan Cancer Hospital and Institute, Sichuan Cancer Centre, School of Medicine, University of Electronic Science and Technology of China, Chengdu, China; ^2^ School of Medicine, University of Electronic Science and Technology of China, Chengdu, China; ^3^ Department of Pharmacy, Dujiangyan People’s Hospital, Dujiangyan Medical Center, Dujiangyan, China; ^4^ Department of Pharmacy, Chengdu Third People’s Hospital, Chengdu, China

**Keywords:** PD-1 inhibitors, PD-L1 inhibitors, systematic review, overview, cancer

## Abstract

**Background:**

An influx of systematic reviews (SRs) of programmed cell death 1 (PD-1) and programmed death ligand-1 (PD-L1) checkpoint inhibitors in cancer treatment with or without meta-analysis and with different methodological quality and inconsistent results have been published, confusing clinical decision making. The aim of this study was to comprehensively evaluate and summarize the current evidence of PD-(L)1 inhibitors in the treatment of cancer.

**Methods:**

A comprehensive search of SRs, which included meta-analyses of PD-(L)1 inhibitors on cancer, was performed on eight databases with a cutoff date of 1 January 2022. Two authors independently identified SRs, extracted data, assessed the report quality according to the guidance of the Preferred Reporting Items for Systematic Reviews and Meta-analyses (PRISMA) statement, evaluated the methodological quality by the Assessment of Multiple Systematic Reviews 2 (AMSTAR 2), and appraised the quality of evidence by the Grading of Recommendations, Assessment, Development, and Evaluation (GRADE).

**Results:**

A total of 172 SRs with meta-analysis met the inclusion criteria. The report quality of included SRs was quite good, with 128 (74.42%) SRs of high quality and 44 (25.58%) of moderate quality. The methodological quality was alarming, as only one (0.58%) SR had high quality, five (2.91%) SRs had low quality, and the other 166 (96.51%) SRs had critically low quality. For GRADE, 38 (3.77%) outcomes had high-quality evidence, 288 (28.57%) moderate, 545 (54.07%) low, and 137 (13.59%) critically low-quality evidence. Current evidence indicated that treatment with PD-(L)1 inhibitors were significantly effective in non-small cell lung cancer, small cell lung cancer, hepatocellular carcinoma, malignant melanoma, renal cell carcinoma, and urothelial carcinoma, breast cancer, and head and neck squamous cell carcinoma with PD-L1 expression level≥1%, whereas the evidence in gastroesophageal and colorectal tumors is still controversial. Monotherapy with PD-(L)1 inhibitors was associated with a lower frequency of any grade and high-grade adverse events (AEs). The incidence of any grade and high-grade AEs caused by PD-(L)1 inhibitors in combination with other therapies was no lower than the controls. However, PD-(L)1 inhibitors were associated with a higher frequency of any grade and high-grade immune-related AEs.

**Conclusions:**

PD-(L)1 inhibitors appeared to be effective and safe for cancer treatment, except for gastrointestinal tumors; however, the quality of the evidence is not convincing. Future studies should improve methodological quality and focus on the sequential trial analysis of subgroups and safety.

**Systematic Review Registration:**

http://www.crd.york.ac.uk/prospero, identifier CRD42020194260.

## Introduction

Cancer is still a devastating disease, representing the main cause of death globally, as well as an important obstacle in improving human life expectancy (1). According to the GLOBOCAN data, there were about 19.3 million new cancer cases and about 10 million new deaths worldwide in 2020 (2). The continuous increase of tumor cases accelerated the development of tumor-related science, while new anti-tumor drugs have been successively developed and marketed. In recent years, programmed cell death 1 (PD-1) and programmed death ligand-1 (PD-L1) checkpoint inhibitors showed obvious developmental potential in treating malignant tumors. Currently, it has become an important research direction for tumor therapy (3, 4).

PD-1 is expressed on the surface of activated T cells, B cells, and other lymphocytes, and its mainly natural ligand is PD-L1. However, PD-L1 is a negative T cell costimulatory molecule, which specifically binds with PD-1 to inhibit T cell activation and reduces the immune effect of T cells, thus having an immunosuppressive role (5). PD-L1 can be continuously expressed on the surface of melanoma, non-small cell lung cancer, breast cancer, and hematological malignancies (6, 7), while PD-(L)1 inhibitors can specifically block the binding of PD-1 and PD-L1 to restore T cell activity and inhibit tumor growth. Since pembrolizumab was approved for unresectable or metastatic melanoma in September 2014 (8, 9), a large number of PD-(L)1 inhibitors have been approved for clinical use (10–18).

Many systematic reviews (SRs) or meta-analyses have reported on the safety and efficacy of PD-(L)1 inhibitors in the treatment of cancer; however, some of these results are inconsistent (19, 20), and the majority of current SRs lack quality control methods such as study registration, protocol, study bias, and publication bias (21, 22). As a result, the overall quality of methodology and evidence is not robust enough, which complicates clinical decision making. Therefore, it is necessary to examine the published SRs of PD-(L)1 inhibitors in the treatment of cancer and identify high-quality evidence so as to provide a reference for clinical application and future clinical studies.

We carried out an overview of SR on the efficacy and safety of PD-1 and PD-L1 inhibitors used as monotherapy or in combination with other therapies. Our aim was to objectively evaluate report quality, methodological quality, and evidence level of reported SRs, and comprehensively analyze qualitative evidence, summarize the evidence on efficacy and safety of PD-(L)1 inhibitors in the treatment of cancer, and screen the best treatment protocols for various types of tumors.

## Methods

### Study Registration

This study strictly followed the Preferred Reporting Items for overview of systematic review (PRIO-harms) (23). We registered a prospective protocol on PROSPERO (NO:CRD42020194260; http://www.crd.york.ac.uk/prospero) (24).

### Search Strategy and Selection Criteria

PubMed, Embase, the Cochrane Library, Web of Science (WOS), China National Knowledge Infrastructure (CNKI), China Science and Technology Journal Database (VIP), Wanfang Data, and China Biology Medicine (CBM) disc were searched from inception to 1 January 2022, with the search terms “PD-1,” “PD-L1,” “Systematic Review,” and “Meta-analysis.” The search strategy was pre-set by the research team, and the final search was performed by a team member (SL Ou). PubMed search strategy is presented in **Table S1**. The references of relevant overviews were also searched so as to avoid omissions. Duplicate studies were removed with Endnote X9.

SRs were included following selection criteria: (i) study: SR with meta-analysis based on random control trial (RCT); (ii) population: malignant tumor patients, regardless of tumor type, stage, age, and gender; (iii) intervention: monotherapy with PD-1/PD-L1 inhibitors or therapy with PD-(L)1 inhibitors in combination with chemotherapy (CT), targeted therapy or other immunotherapy; (iv) control: non–PD-1/PD-L1 inhibitors or investigator chosen CT; (v) outcomes: progression-free survival (PFS), overall survival (OS), objective response rate (ORR), any grade and high-grade (≥3 grade) adverse events (AEs), and any grade and high-grade (≥3 grade) immune-related AEs (irAEs). Exclusion criteria included the following: (i) repeated studies, (ii) studies written in languages other than English or Chinese, (iii) conference abstracts, (iv) SR not based on RCT, and (v) SR without meta-analysis.

### Data Extraction

Data from included SRs were independently extracted by two investigators (JL and SL Ou). Extraction contents included (i) basic information: study name, first author, country, publication date, numbers of sample size, and type of tumors; (ii) methodological information: registration number, intervention measures, control measures, and methodological tools; (iii) outcome indicators: hazard ratio (HR), odds ratio (OR), and relative risk (RR) of PFS, OS, ORR, AEs, and irAEs.

### Study Quality Assessment

Two investigators (HW and SL Ou) independently evaluated whether the 27 items of the Preferred Reporting Items for Systematic Reviews and Meta-analyses (PRISMA) statement, including title, abstract, introduction, methods, results, discussion, and funding, were reported (25, 26). Any conflicts were resolved by a third author (XL Qin). The evaluation principle of each item was as follows: 1 point represents a report, 0.5 point represents a partial report, and 0 point represents no report. The report quality was judged according to the total score of each systematic review (≥22 is high quality, 11–21 is moderate quality, and ≤10 is low quality).

Two investigators (HW and SL Ou) independently assessed the methodological quality of included SRs by the Measurement Tool to Assess Systematic Reviews 2 (AMSTAR 2) (27). The AMSTAR 2 tool contains 16 items, among which items 2, 4, 7, 9, 11, 13, and 15 are the critical items. Methodological results are classified as high quality (no/only one non-critical weakness), moderate quality (more than one non-critical item weakness), low quality (one critical flaw with or without non-critical weaknesses), and critically low quality (more than one critical flaw with or without non-critical weaknesses).

The Grades of Recommendations, Assessment, Development, and Evaluation (GRADE) tool recommended by the Cochrane Collaboration was used to assess the quality of the evidence of outcome indicators (28). Evidence based on RCT is generally defined as high-quality evidence. Although it is theoretically feasible to improve the quality of evidence of RCT results, there is no convincing study so far (29), so we focused on evidence degradation factors. The degradation factors were (i) risk of bias (RoB) including no allocation concealment, no blind method for participants, selective reporting, no intentional analysis, and incomplete outcome data; (ii) inconsistency (there is heterogeneity in results and the researchers failed to realize and give reasonable explanation); (iii) indirectness (two interventions were compared through another); (iv) imprecision (the smaller sample size included in the study leads to wider confidence intervals); and (v) publication bias (publication bias may exist when the funnel plot is asymmetric, there is a small number of included studies and the results are positive, or the included studies are all sponsored by enterprises). The quality of evidence was not downgraded to “high quality”,while 1 meant it was downgraded to “moderate quality,” 2 meant it was downgraded to “low quality,” and ≥3 meant it was downgraded to “critically low quality.”

## Results

### Study Selection and Characteristics

**Figure 1:**
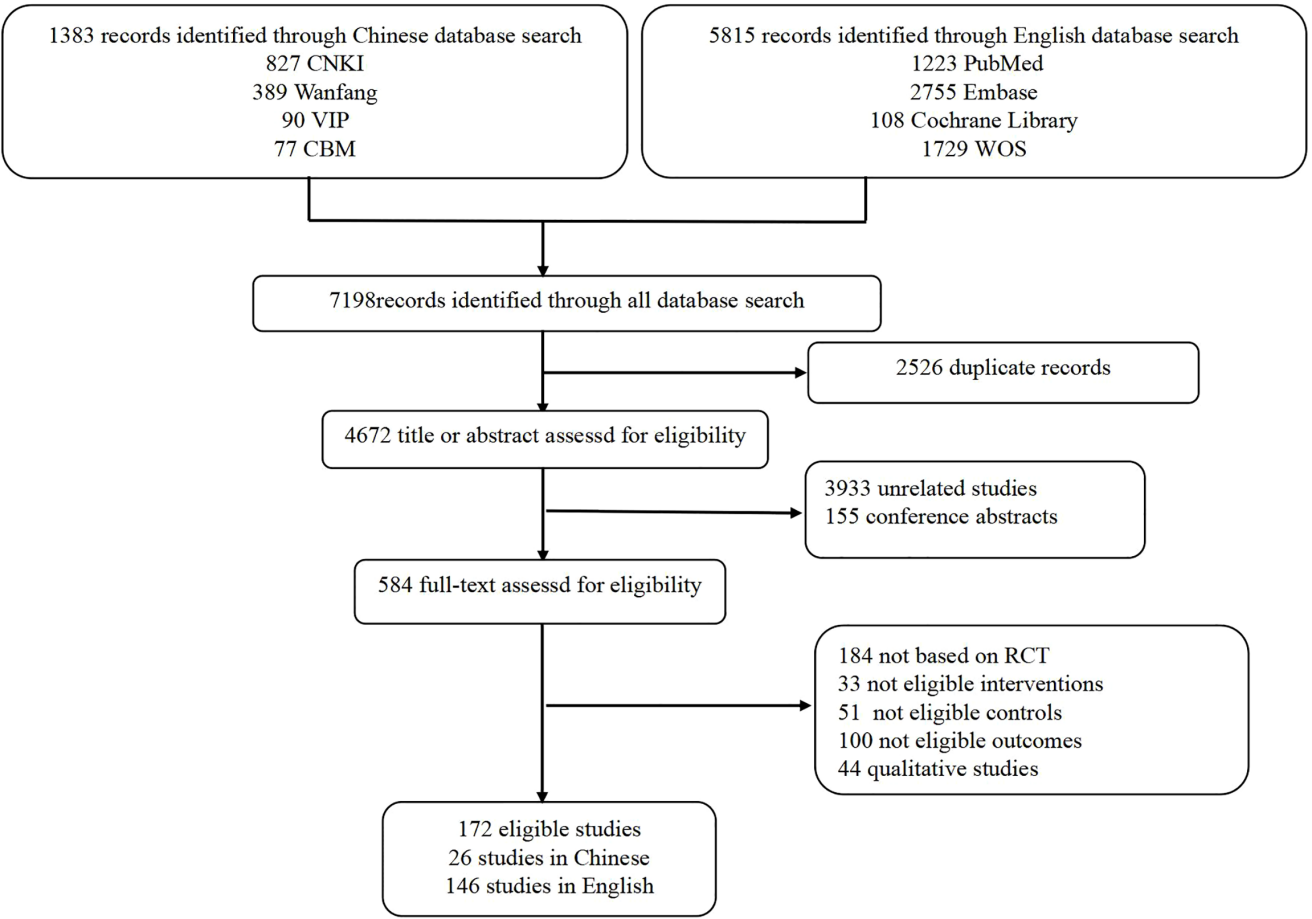
Flow chart of study selection.

Among the 7,198 records, 172 SRs with meta-analysis published between 2015 and 2021 were identified, 146 of which were in English and 26 were in Chinese (**Figure 1**). The number of included RCTs ranged from 2 to 41, and the sample size ranged from 467 to 24902. Moreover, 112 SRs assessed the methodological quality of the included RCTs based on the Cochrane Risk of Bias tool, 30 SRs based on the Jadad scale, one SR based on the Final Delphi List, one SR based on the Method for Evaluating Research and Guideline Evidence tool, whereas 28 SRs did not specify methodological tool. The main subjects were non-small cell lung cancer (NSCLC; *n* = 76), renal cell carcinoma (RCC; *n* = 17), gastroesophageal cancer (*n* = 11), and metastatic melanoma (MM; *n* = 10). More characteristics are presented in **Table S2**.

### Report Quality of Included Systematic Reviews

According to the results assessed by the PRISMA statement, the overall report quality of included SRs was quite good. A total of 128 (74.42%) SRs were of high quality, whereas the remaining ones were of moderate quality. The mainly missing items were “protocol and registration,” “study selection,” and “risk of bias across studies.” The most serious issue was that only 19 (11.05%) SRs reported “protocol and registration.” Information on reported and missing items is shown in **Table 1** and **Table S3**.

**Table 1:** Report quality of included studies.

Section/topic	Item no.	Checklist item	Number of studies reported	Ratio (%)
Title	1	Title	172	100
Abstract	2	Structured summary	149	86.63
Introduction	3	Rationale	172	100
	4	Objectives	172	100
Methods	5	Protocol and registration	19	11.05
	6	Eligibility criteria	170	98.84
	7	Information sources	172	100
	8	Search	172	100
	9	Study selection	98	56.98
	10	Data collection process	164	95.35
	11	Data items	168	97.67
	12	Risk of bias in individual studies	135	78.49
	13	Summary measures	169	98.26
	14	Synthesis of results	172	100
	15	Risk of bias across studies	93	54.07
	16	Additional analyses	105	61.05
Results	17	Study selection	169	98.26
	18	Study characteristics	172	100
	19	Risk of bias within studies	140	81.4
	20	Results of individual studies	172	100
	21	Synthesis of results	172	100
	22	Risk of bias across studies	97	56.4
	23	Additional analysis	146	84.88
Discussion	24	Summary of evidence	172	100
	25	Limitations	144	83.72
	26	Conclusions	172	100
Funding	27	Funding	92	53.49

### The Methodological Quality of Included Systematic Reviews

The results of AMSTAR 2 are presented in **Table S4**. Due to the serious critical items missing, only one (0.58%) study was rated as high quality, five (2.91%) studies as low quality, and the other 166 (96.51%) studies as critically low quality. For the critical items, appropriate methods for statistical combination of results were well done in all included SRs (item 11), and comprehensive search strategy were well done in 159 (92.44%) SRs (item 4), whereas merely 19 (11.05%) SRs reported predefined protocol (item 2) and just two (1.16%) SRs provided a list of excluded RCTs (item 7), resulting in most of the studies having critically low quality. The evaluation of the RoB was also seriously missing, considering that 28 (16.28%) SRs did not report the RoB of included RCTs (item 9) and 125 (72.67%) SRs did not account for RoB when interpreting the results (item 13). For noncritical items, the absence of items was equally pervasive except for study designs for inclusion (item 3) and the detailed characteristics of included studies (item 8), which were reported in all SRs. Also, 157 (91.28%) SRs included the components of PICO (population, intervention, control group, outcome) of the research question and inclusion criteria (item 1). In addition, 85 (49.42%) SRs performed study selection (item 5), and in 135 (78.49%) SRs, data extractions (item 6) were independently performed by two investigators. As for research conflicts of interest, 132 (76.74%) SRs reported possible conflicts of interest (item 16), and only 99 (57.56%) SRs reported funding for the study (item 10). Besides, only 40 (23.26%) SRs assessed the potential effect of RoB on the evidence synthesis (item 12), and 111 (64.53%) SRs explained the possible reasons for heterogeneity in the results (item 14).

### Quality of Evidence of SRs

A total of 1,008 outcomes of the included 172 SRs were appraised by GRADE (**Table S5**). The results evaluated by the GRADE tool showed that 38 (3.77%) of them were of high quality, 288 (28.57%) of moderate quality, 545 (54.07%) of low quality, and 137 (13.59%) of critically low quality. As we only considered comparisons between interventions and controls, no outcomes were downgraded for indirectness. Also, all the sample sizes of included SRs were >200. We found that only nine subgroup analyses results were downgraded for imprecision. Nevertheless, because partial RCTs did not state allocation concealment and blinding of outcome assessment, high heterogeneity of synthesis of results, and publication bias, most outcomes were assessed as low quality or critically low quality.

### Efficacy and Safety of *Programmed Death (Ligand)-1* Inhibitors in the Treatment of Cancer

**Figure 2:**
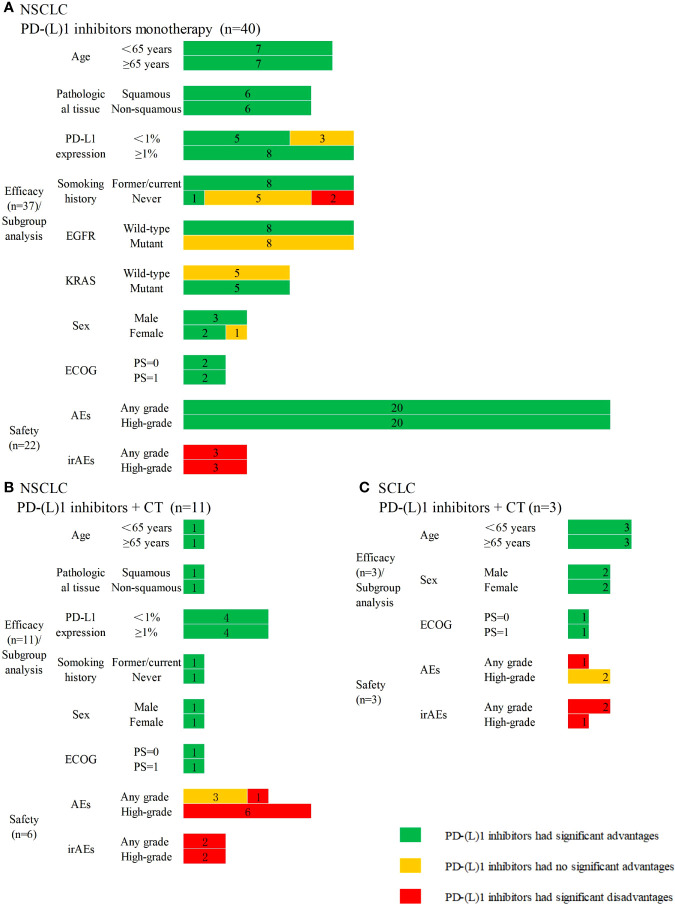
Subgroup analyses and safety results in lung cancer. **(A)** PD-(L)1 inhibitors monotherapy versus chemotherapy in non-small cell lung cancer; **(B)** PD-(L)1 inhibitors plus chemotherapy versus chemotherapy in non-small cell lung cancer; **(C)** PD-(L)1 inhibitors plus chemotherapy versus chemotherapy in small cell lung cancer. CT, chemotherapy; n, the number of included systematic reviews; EFGR, epidermal growth factor receptor; KRAS, kirsten rat sarcoma viral oncogene; ECOG, Eastern Cooperative Oncology Group; PS, performance status; AEs, adverse events; irAEs, immune-related adverse events; High-grade, ≥3 grade.

A total of 37 SRs assessed the efficacy, and 22 SRs assessed the safety of PD-(L)1 inhibitors used as monotherapy compared with CT in NSCLC. Also, 36 SRs indicated PD-(L)1 inhibitors significantly improved the efficacy, except one SR reporting no clinical survival benefit with nivolumab versus CT, even in patients with levels of PD-L1 expression level≥5%. Subgroup analyses and safety results are presented in **Figure 2A**. Eleven SRs assessed the efficacy, and six SRs assessed the safety of PD-(L)1 inhibitors plus CT versus CT in the treatment of NSCLC. All 11 SRs indicated that PD-(L)1 inhibitors plus CT significantly improved the efficacy. Subgroup analyses and safety results are presented in **Figure 2B**.

Three SRs assessed the efficacy and safety of PD-(L)1 inhibitors plus CT compared with CT in the treatment of SCLC. All three SRs indicated that PD-(L)1 plus CT significantly improved the efficacy. Subgroup analyses and safety results are presented in **Figure 2C**.

**Figure 3:**
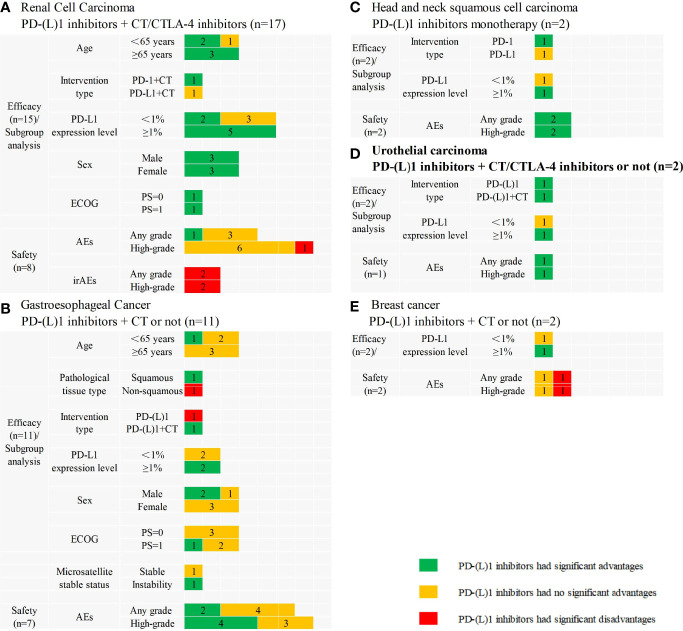
Subgroup analyses and safety results in other cancers. **(A)** PD-(L)1 inhibitors plus chemotherapy/cytotoxic T lymphocyte associated antigen 4 pathway inhibitors versus chemotherapy in renal cell carcinoma; **(B)** PD-(L)1 inhibitors plus chemotherapy or not versus chemotherapy in gastroesophageal cancer; **(C)** PD-(L)1 inhibitors monotherapy versus chemotherapy in head and neck squamous cell carcinoma; **(D)** PD-(L)1 inhibitors plus chemotherapy/cytotoxic T lymphocyte associated antigen 4 pathway inhibitors or not versus chemotherapy in urothelial carcinoma; **(E)** PD-(L)1 inhibitors plus chemotherapy or not versus chemotherapy in breast cancer. CT, chemotherapy; CTLA-4, cytotoxic T lymphocyte associated antigen 4; n, the number of included systematic reviews; ECOG, Eastern Cooperative Oncology Group; PS, performance status; AEs, adverse events; irAEs, immune-related adverse events; High-grade, ≥3 grade.

Fifteen SRs assessed the efficacy and eight SRs assessed the safety of PD-(L)1 inhibitors in combination with CT or cytotoxic T lymphocyte-associated antigen-4 (CTLA-4) compared with CT in treating RCC. All 15 SRs indicated that interventions significantly improved the efficacy, and 11 SRs confirmed that sunitinib monotherapy no longer represented the first-line treatment for metastatic RCC patients. Subgroup analyses and safety results are presented in **Figure 3A**.

A total of 11 SRs assessed the efficacy of PD-(L)1 inhibitors compared with CT in the treatment of Gastroesophageal Cancer, two of which integrated PD-(L)1 inhibitors monotherapy and PD-(L)1 inhibitors plus CT, whereas others used PD-(L)1 inhibitors as monotherapy, and seven SRs assessed the safety of PD-(L)1 inhibitors monotherapy. The efficacy results were inconsistent, as eight SRs indicated PD-(L)1 inhibitors had significant advantages in OS or PFS, and three SRs indicated that PD-(L)1 inhibitors did not significantly differ with CT. Subgroup analyses and safety results are presented in **Figure 3B**.

Two SRs assessed the efficacy and safety of PD-(L)1 inhibitors monotherapy versus CT in treating head and neck squamous cell carcinoma. All SRs indicated PD-(L)1 inhibitors had significant advantages in OS. Subgroup analyses and safety results are presented in **Figure 3C**.

Two SRs assessed the efficacy, and one SR assessed the safety of PD-(L)1 inhibitors monotherapy integrated PD-(L)1 inhibitors plus CT or CTLA-4 inhibitors in treating urothelial carcinoma. The results are presented in **Figure 3D**.

One SRs assessed the efficacy and two SRs assessed the safety of PD-(L)1 inhibitors plus CT or not in treating breast cancer. The results indicated PD-(L)1 inhibitors plus CT did not significantly differ from CT in OS. Subgroup analysis and safety results are presented in **Figure 3E**.

Four SRs assessed the efficacy, and five SRs assessed the safety of PD-1 inhibitors used as monotherapy versus CT or CTLA-4 inhibitors in the treatment of MM. All four SRs indicated PD-1 inhibitors monotherapy significantly improved PFS, ORR, or OS. Since the controls integrated CT and CTLA-4 inhibitors, the results of AEs were inconsistent; three SRs indicated that PD-1 inhibitors were associated with lower frequency of any grade AEs whereas two SRs indicated no significant difference. Five SRs assessed the efficacy, and two SRs assessed the safety of PD-1 inhibitor monotherapy that integrated PD-1 inhibitors plus CTLA-4 inhibitors in the treatment of MM. All five SRs indicated that PD-1 inhibitors groups significantly improved efficacy. For the safety, one SR indicated no significant difference between interventions and controls in any grade AEs and high-grade AEs. One SR analyzed subgroups by the type of interventions, indicating that PD-1 inhibitor monotherapy significantly reduced the reactions of any grade AEs versus CT or CTLA-4 inhibitors, whereas PD-1 inhibitor plus CTLA-4 inhibitors were significantly associated with high-grade AEs.

One SR assessed the efficacy and safety of PD-(L)1 inhibitors in treating hepatocellular carcinoma. The results showed that when compared with CT, PD-(L)1 inhibitors combined with CT or not significantly improved PFS, OS, and ORR, and reduced the reactions of high-grade AEs.

One SR integrated PD-1 inhibitor monotherapy and PD-1 inhibitors plus CTLA-4 inhibitors in the treatment of colorectal cancer, showing that PD-1 inhibitors groups had no significant difference in ORR, any grade AEs and high-grade AEs.

Moreover, 44 SRs did not specify the types of tumors, and three SRs did not specify the pathological tissue type of lung cancer, as shown in **Table S5**.

## Discussion

### Summary of Results

This overview included 172 SRs based on RCT published in English and Chinese, which were used to evaluate and summarize evidence on the safety and efficacy of PD-(L)1 in cancer treatment. The current evidence indicated significant advantageous efficacy in NSCLC, SCLC, hepatocellular carcinoma, MM, RCC, and urothelial carcinoma, breast cancer, and head and neck squamous cell carcinoma with PD-L1 expression level≥1% treated with PD-(L)1 inhibitors, whereas the results in gastroesophageal and colorectal tumors were controversial. In the subgroup analyses of NSCLC, partial evidence significantly improved efficacy in patients with negative PD-L1 expression, thus providing treatment options for the patients with PD-L1 <1%. In the epidermal growth factor receptor (EGFR) mutant and kirsten rat sarcoma viral oncogene (KRAS) wild-type population, evidence indicated that PD-(L)1 inhibitors did not respond according to the latest research; this may be related to the tumor microenvironment, as EGFR mutant and KRAS wild-type tumors are associated with a high frequency of inactive tumor-infiltrating lymphocytes (30, 31). For never smokers, more evidence indicated that PD-(L)1 inhibitors did not respond, and all evidence indicated that former/current smokers had significant advantages, which may be because smoking causes a greater tumor mutation burden (32). In the subgroup analyzed by PD-L1 expression level, we found significantly improved efficacy only in patients with PD-L1≥1% but not with PD-L1<1% in the treatment of urothelial carcinoma, breast cancer, and head and neck squamous cell carcinoma, which suggested that PD-L1 expression level may be used as a biomarker (33–35).

In general, monotherapy with PD-(L)1 inhibitors was associated with lower frequency of any grade and high-grade AEs. The incidence of any grade and high-grade AEs of PD-(L)1 inhibitors in combination with other therapies was not lower compared with controls; however, it is necessary to pay attention to the occurrence of any grade of irAEs and high-grade irAEs associated with PD-(L)1 inhibitors.

Unfortunately, only one Cochrane SR was rated as high quality according to AMSTAR 2, whereas the others were of low or critically low quality. The main reasons for this were a poor practice of the critical items 2 and 7. As in observational research, a systematic review should adhere to a well-developed protocol before the beginning of the study so as to reduce the RoB and duplicate studies (27); however, only 11.05% (19 of 172) SRs reported predefined protocol, and we found that the content of a large number of included SRs was repeated, which had the same PICOS, and their retrieval results greatly differed, which may be due to the lack of study protocols, resulting in serious resource waste and evidence interference. Although all included SRs provided flow diagrams of the studies’ search and selection, just 1.16% (two of 172) SRs provided a list of excluded studies, which led to selection bias. Also, we found that 76.74% (132 of 172) SRs did not assess the potential impact of RoB on the results of the evidence synthesis, and 72.67% (125 of 172) SRs did not account for RoB in primary RCTs when interpreting the evidence synthesis, which indicated that a large proportion of included SRs did not objectively state the authenticity of the results. Thus, we expect future SRs to focus more on these items to improve the methodological quality.

When it comes to quality of evidence, we found only 3.77% (38 of 1008) outcomes of high quality, 28.57% (288 of 1008) of moderate quality, 54.07% (545 of 1008) of low quality, and 13.59% (137 of 1008) of critically low quality. The main degradation factors were RoB (797 of 1008) and publication bias (772 of 1008). Our study found that evidence of SRs was degraded due to RoB on missing allocation concealment and blinding of outcome assessment in partially included RCTs, which had a great influence on the results, especially the subjective results such as ORR and AEs (36). The demotions due to publication bias were mainly due to the small number of subgroup analysis studies. Since RCTs were recruited with predetermined patients and the subgroup analyses were not necessarily consistent across SRs, the results of subgroup analyses should be further verified.

### Implications

This overview has some implications. First, this overview showed inconsistent results in gastrointestinal tumors and subgroup analysis of some tumors, especially in the subgroup of PD-L1 expression level. Thus, future studies should apply trial sequential analysis (TSA) to evaluate whether the current evidence is sufficient or if further clinical trials are needed. Second, as AEs are not usually the objective of clinical trials, statistical sample sizes for calculating the incidence of AEs may be inaccurate. We found that only one SR established sufficient and conclusive evidence of fatal AEs associated with PD-(L)1 monotherapy, thus indicating that future studies should focus on cumulative meta-analysis or TSA of AEs reaction to PD-(L)1 inhibitors (37). Third, we found that the overall methodological quality of included SRs was critically low, causing the loss of the guiding clinical significance. In addition, many unregistered duplicate SRs also caused evidence-based reference interference, thus suggesting that subsequent systematic review reports should comply with the requirements of the PRISMA statement and AMSTAR 2 methodology tool. Fourth, most of the included SRs in this overview missed the subgroup of which drugs that PD-(L)1 inhibitors for monotherapy and in combination with CT, thus indicating that future systematic reviews and meta-analyses should focus on this area to provide accurate evidence-based evidence for clinical use.

### Strengths and Limitations

This overview summarized the current evidence about the PD-(L)1 inhibitors in cancer treatment, classified the results by different types of cancer, and assessed the methodological quality with AMSTAR 2 and the quality of evidence with GRADE. Nonetheless, the present study has several limitations. First, this overview was limited by the characteristics and methodological quality of the included SRs, as the absence of SRs data may affect our judgment to some extent. Second, the AMSTAR 2 and GRADE tools are highly subjective. Although we strictly followed the double cross-check method, it is impossible to avoid the occurrence of bias. Third, the vast majority of SRs methodologies included in this overview were of low or critically low value, which seriously affected the accuracy of the results. Finally, linguistic bias is inevitable, since we only included Chinese and English SRs.

## Conclusions

Our results revealed that the treatment with PD-(L)1 inhibitors had significant efficacy in NSCLC, SCLC, hepatocellular carcinoma, MM, RCC, and urothelial carcinoma, breast cancer, and head and neck squamous cell carcinoma with PD-L1 expression level≥1%, whereas evidence in gastroesophageal and colorectal tumors was controversial. Also, more attention should be paid to the occurrence of irAEs. However, the methodological quality and quality of evidence of many SRs were low or critically low. Future studies should improve methodological quality and focus on TSA of subgroups, including safety.

## Data Availability Statement

The original contributions presented in the study are included in the article/**Supplementary Material**. Further inquiries can be directed to the corresponding author.

## Author Contributions

QJ and S-LO contributed to the study design. JL, HW, X-LQ, and S-LO contributed to literature retrieval and data collection and evaluation. S-YD and SW critically revised the manuscript. All authors read and approved the final manuscript.

## Funding

This work was supported by the Fundamental Research Funds for the Central Universities of China (grant number ZYGX2021J038) and the Medical Science and Technology Project of the Sichuan Provincial Health Commission (grant number 21PJ115). The funders had no role in the study design, data collection and analysis, decision to publish, or manuscript preparation.

## Conflict of Interest

The authors declare that the research was conducted in the absence of any commercial or financial relationships that could be construed as a potential conflict of interest.

## Publisher’s Note

All claims expressed in this article are solely those of the authors and do not necessarily represent those of their affiliated organizations, or those of the publisher, the editors and the reviewers. Any product that may be evaluated in this article, or claim that may be made by its manufacturer, is not guaranteed or endorsed by the publisher.

## References

[1] Bray F Laversanne M Weiderpass E Soerjomataram I . The Ever-Increasing Importance of Cancer as a Leading Cause of Premature Death Worldwide. Cancer (2021) 127(16):3029–30. doi: 10.1002/cncr.33587 34086348

[2] Sung H Ferlay J Siegel RL Laversanne M Soerjomataram I Jemal A . Global Cancer Statistics 2020: Globocan Estimates of Incidence and Mortality Worldwide for 36 Cancers in 185 Countries. CA Cancer J Clin (2021) 71(3):209–49. doi: 10.3322/caac.21660 33538338

[3] Postow MA Callahan MK Wolchok JD . Immune Checkpoint Blockade in Cancer Therapy. J Clin Oncol (2015) 33(17):1974–82. doi: 10.1200/jco.2014.59.4358 PMC4980573 25605845

[4] Ribas A Wolchok JD . Cancer Immunotherapy Using Checkpoint Blockade. Science (New York NY) (2018) 359(6382):1350–5. doi: 10.1126/science.aar4060 PMC7391259 29567705

[5] Amarnath S Mangus CW Wang JC Wei F He A Kapoor V . The Pdl1-Pd1 Axis Converts Human Th1 Cells Into Regulatory T Cells. Sci Trans Med (2011) 3(111):111ra20. doi: 10.1126/scitranslmed.3003130 PMC3235958 22133721

[6] Liu J Hamrouni A Wolowiec D Coiteux V Kuliczkowski K Hetuin D . Plasma Cells From Multiple Myeloma Patients Express B7-H1 (Pd-L1) and Increase Expression After Stimulation With Ifn-{Gamma} and Tlr Ligands *Via* a Myd88-, Traf6-, and Mek-Dependent Pathway. Blood (2007) 110(1):296–304. doi: 10.1182/blood-2006-10-051482 17363736

[7] Cortes J Cescon DW Rugo HS Nowecki Z Im SA Yusof MM . Pembrolizumab Plus Chemotherapy Versus Placebo Plus Chemotherapy for Previously Untreated Locally Recurrent Inoperable or Metastatic Triple-Negative Breast Cancer (Keynote-355): A Randomised, Placebo-Controlled, Double-Blind, Phase 3 Clinical Trial. Lancet (London England) (2020) 396(10265):1817–28. doi: 10.1016/s0140-6736(20)32531-9 33278935

[8] Poole RM . Pembrolizumab: First Global Approval. Drugs (2014) 74(16):1973–81. doi: 10.1007/s40265-014-0314-5 25331768

[9] Ribas A Puzanov I Dummer R Schadendorf D Hamid O Robert C . Pembrolizumab Versus Investigator-Choice Chemotherapy for Ipilimumab-Refractory Melanoma (Keynote-002): A Randomised, Controlled, Phase 2 Trial. Lancet Oncol (2015) 16(8):908–18. doi: 10.1016/s1470-2045(15)00083-2 PMC9004487 26115796

[10] Robert C Long GV Brady B Dutriaux C Maio M Mortier L . Nivolumab in Previously Untreated Melanoma Without Braf Mutation. N Engl J Med (2015) 372(4):320–30. doi: 10.1056/NEJMoa1412082 25399552

[11] Keam SJ . Toripalimab: First Global Approval. Drugs (2019) 79(5):573–8. doi: 10.1007/s40265-019-01076-2 30805896

[12] Hoy SM . Sintilimab: First Global Approval. Drugs (2019) 79(3):341–6. doi: 10.1007/s40265-019-1066-z 30742278

[13] Markham A Keam SJ . Camrelizumab: First Global Approval. Drugs (2019) 79(12):1355–61. doi: 10.1007/s40265-019-01167-0 31313098

[14] Lee A Keam SJ . Tislelizumab: First Approval. Drugs (2020) 80(6):617–24. doi: 10.1007/s40265-020-01286-z 32185681

[15] Markham A Duggan S . Cemiplimab: First Global Approval. Drugs (2018) 78(17):1841–6. doi: 10.1007/s40265-018-1012-5 30456447

[16] Syed YY . Durvalumab: First Global Approval. Drugs (2017) 77(12):1369–76. doi: 10.1007/s40265-017-0782-5 PMC5636860 28643244

[17] Markham A . Atezolizumab: First Global Approval. Drugs (2016) 76(12):1227–32. doi: 10.1007/s40265-016-0618-8 27412122

[18] Kim ES . Avelumab: First Global Approval. Drugs (2017) 77(8):929–37. doi: 10.1007/s40265-017-0749-6 28456944

[19] Kundel Y Sternschuss M Moore A Perl G Brenner B Goldvaser H . Efficacy of Immune-Checkpoint Inhibitors in Metastatic Gastric or Gastroesophageal Junction Adenocarcinoma by Patient Subgroups: A Systematic Review and Meta-Analysis. Cancer Med (2020) 9(20):7613–25. doi: 10.1002/cam4.3417 PMC7571828 32869544

[20] Maoxi Z Jinmin X Xiaozhu Z Yubing Y Yuxi Z . Pd-1/Pd-L1 Inhibitors Versus Chemotherapy for Previously Treated Advanced Gastroesophageal Cancer: A Meta-Analysis of Randomized Controlled Trials. J Oncol (2021) 2021:3048974. doi: 10.1155/2021/3048974 34567113 PMC8463210

[21] Lee CK Man J Lord S Cooper W Links M Gebski V . Clinical and Molecular Characteristics Associated With Survival Among Patients Treated With Checkpoint Inhibitors for Advanced Non-Small Cell Lung Carcinoma: A Systematic Review and Meta-Analysis. JAMA Oncol (2018) 4(2):210–6. doi: 10.1001/jamaoncol.2017.4427 PMC5838598 29270615

[22] Jiang T Liu H Qiao M Li X Zhao C Su C . Impact of Clinicopathologic Features on the Efficacy of Pd-1/Pd-L1 Inhibitors in Patients With Previously Treated Non-Small-Cell Lung Cancer. Clin Lung Cancer (2018) 19(2):e177–e84. doi: 10.1016/j.cllc.2017.10.018 29175386

[23] Bougioukas KI Liakos A Tsapas A Ntzani E Haidich AB . Preferred Reporting Items for Overviews of Systematic Reviews Including Harms Checklist: A Pilot Tool to Be Used for Balanced Reporting of Benefits and Harms. J Clin Epidemiol (2018) 93:9–24. doi: 10.1016/j.jclinepi.2017.10.002 29037888

[24] Booth A Clarke M Ghersi D Moher D Petticrew M Stewart L . An International Registry of Systematic-Review Protocols. Lancet (London England) (2011) 377(9760):108–9. doi: 10.1016/s0140-6736(10)60903-8 20630580

[25] Moher D Liberati A Tetzlaff J Altman DG . Preferred Reporting Items for Systematic Reviews and Meta-Analyses: The Prisma Statement. BMJ (Clinical Res ed) (2009) 339:b2535. doi: 10.1136/bmj.b2535 PMC2714657 19622551

[26] Liberati A Altman DG Tetzlaff J Mulrow C Gøtzsche PC Ioannidis JP . The Prisma Statement for Reporting Systematic Reviews and Meta-Analyses of Studies That Evaluate Health Care Interventions: Explanation and Elaboration. Ann Intern Med (2009) 151(4):W65–94. doi: 10.7326/0003-4819-151-4-200908180-00136 19622512

[27] Shea BJ Reeves BC Wells G Thuku M Hamel C Moran J . Amstar 2: A Critical Appraisal Tool for Systematic Reviews That Include Randomised or Non-Randomised Studies of Healthcare Interventions, or Both. BMJ (Clinical Res ed) (2017) 358:j4008. doi: 10.1136/bmj.j4008 PMC5833365 28935701

[28] Guyatt GH Oxman AD Vist GE Kunz R Falck-Ytter Y Alonso-Coello P . Grade: An Emerging Consensus on Rating Quality of Evidence and Strength of Recommendations. BMJ (Clinical Res ed) (2008) 336(7650):924–6. doi: 10.1136/bmj.39489.470347.AD PMC2335261 18436948

[29] Guyatt GH Oxman AD Sultan S Glasziou P Akl EA Alonso-Coello P . Grade Guidelines: 9. Rating Up the Quality of Evidence. J Clin Epidemiol (2011) 64(12):1311–6. doi: 10.1016/j.jclinepi.2011.06.004 21802902

[30] Rizvi NA Hellmann MD Snyder A Kvistborg P Makarov V Havel JJ . Cancer Immunology. Mutational Landscape Determines Sensitivity to Pd-1 Blockade in Non-Small Cell Lung Cancer. Science (New York NY) (2015) 348(6230):124–8. doi: 10.1126/science.aaa1348 PMC4993154 25765070

[31] Skoulidis F Byers LA Diao L Papadimitrakopoulou VA Tong P Izzo J . Co-Occurring Genomic Alterations Define Major Subsets of Kras-Mutant Lung Adenocarcinoma With Distinct Biology, Immune Profiles, and Therapeutic Vulnerabilities. Cancer Discov (2015) 5(8):860–77. doi: 10.1158/2159-8290.Cd-14-1236 PMC4527963 26069186

[32] Govindan R Ding L Griffith M Subramanian J Dees ND Kanchi KL . Genomic Landscape of Non-Small Cell Lung Cancer in Smokers and Never-Smokers. Cell (2012) 150(6):1121–34. doi: 10.1016/j.cell.2012.08.024 PMC3656590 22980976

[33] Suzman DL Agrawal S Ning YM Maher VE Fernandes LL Karuri S . Fda Approval Summary: Atezolizumab or Pembrolizumab for the Treatment of Patients With Advanced Urothelial Carcinoma Ineligible for Cisplatin-Containing Chemotherapy. Oncologist (2019) 24(4):563–9. doi: 10.1634/theoncologist.2018-0084 PMC6459239 30541754

[34] Schmid P Adams S Rugo HS Schneeweiss A Barrios CH Iwata H . Atezolizumab and Nab-Paclitaxel in Advanced Triple-Negative Breast Cancer. N Engl J Med (2018) 379(22):2108–21. doi: 10.1056/NEJMoa1809615 30345906

[35] Hsu C Lee SH Ejadi S Even C Cohen RB Le Tourneau C . Safety and Antitumor Activity of Pembrolizumab in Patients With Programmed Death-Ligand 1-Positive Nasopharyngeal Carcinoma: Results of the Keynote-028 Study. J Clin Oncol (2017) 35(36):4050–6. doi: 10.1200/jco.2017.73.3675 28837405

[36] Wood L Egger M Gluud LL Schulz KF Jüni P Altman DG . Empirical Evidence of Bias in Treatment Effect Estimates in Controlled Trials With Different Interventions and Outcomes: Meta-Epidemiological Study. BMJ (Clinical Res ed) (2008) 336(7644):601–5. doi: 10.1136/bmj.39465.451748.AD PMC2267990 18316340

[37] Zhao B Zhao H Zhao J . Fatal Adverse Events Associated With Programmed Cell Death Protein 1 or Programmed Cell Death-Ligand 1 Monotherapy in Cancer. Ther Adv Med Oncol (2020) 12:1758835919895753. doi: 10.1177/1758835919895753 32082425 PMC7005982

